# Development and use of a flexible data harmonization platform to facilitate the harmonization of individual patient data for meta-analyses

**DOI:** 10.1186/s13104-019-4210-7

**Published:** 2019-03-22

**Authors:** Joeri Kalter, Maike G. Sweegers, Irma M. Verdonck-de Leeuw, Johannes Brug, Laurien M. Buffart

**Affiliations:** 1 Department of Epidemiology and Biostatistics, Amsterdam University Medical Centres, Vrije Universiteit Amsterdam, De Boelelaan 1089a, 1081 HV Amsterdam, The Netherlands; 2 Amsterdam Public Health Research Institute, Amsterdam, The Netherlands; 3 Cancer Center Amsterdam, Amsterdam, The Netherlands; 4 Department of Otolaryngology-Head and Neck Surgery, Amsterdam University Medical Centres, Vrije Universiteit Amsterdam, Amsterdam, The Netherlands; 5 Department of Clinical- Developmental- and Neuro Psychology, Faculty of Behavioral and Movement Sciences, Vrije Universiteit Amsterdam, Amsterdam, The Netherlands; 6 Amsterdam School of Communication Research (ASCoR), University of Amsterdam, Amsterdam, The Netherlands; 7 Department of Medical Oncology, Amsterdam University Medical Centres, Vrije Universiteit Amsterdam, Amsterdam, The Netherlands

**Keywords:** Flexible data harmonization platform, Easy in use infrastructure, Centralized and secured database server

## Abstract

**Objective:**

Harmonizing individual patient data (IPD) for meta-analysis has clinical and statistical advantages. Harmonizing IPD from multiple studies may benefit from a flexible data harmonization platform (DHP) that allows harmonization of IPD already during data collection. This paper describes the development and use of a flexible DHP that was initially developed for the Predicting OptimaL cAncer RehabIlitation and Supportive care (POLARIS) study.

**Results:**

The DHP that we developed (I) allows IPD harmonization with a flexible approach, (II) has the ability to store data in a centralized and secured database server with large capacity, (III) is transparent and easy in use, and (IV) has the ability to export harmonized IPD and corresponding data dictionary to a statistical program. The DHP uses Microsoft Access as front-end application and requires a relational database management system such as Microsoft Structured Query Language (SQL) Server or MySQL as back-end application. The DHP consists of five user friendly interfaces which support the user to import original study data, to harmonize the data with a master data dictionary, and to export the harmonized data into a statistical software program of choice for further analyses. The DHP is now also adopted in two other studies.

**Electronic supplementary material:**

The online version of this article (10.1186/s13104-019-4210-7) contains supplementary material, which is available to authorized users.

## Introduction

Meta-analyses that synthesize results from multiple studies inform health professionals about the best available care and are an essential part of evidence-based medicine [1, 2]. A meta-analysis on individual patient data (IPD) is regarded as the gold standard for meta-analysis [3] because it allows standardized analytical techniques across studies, the testing of interaction effects with covariates at the level of the patient, and the use of consistent analyses for time-to-event outcomes [4, 5].

Gathering and harmonizing IPD from individual studies is dependent on response of principal investigators (PI’s) from the original study, their time to prepare their data for data sharing, or on a study’s privacy, ethical or legal issues [6]. Additionally, researchers conducting the IPD meta-analysis may face difficulties with harmonizing IPD because different studies often used different coding schemes or constructs [7].

**Fig. 1:**
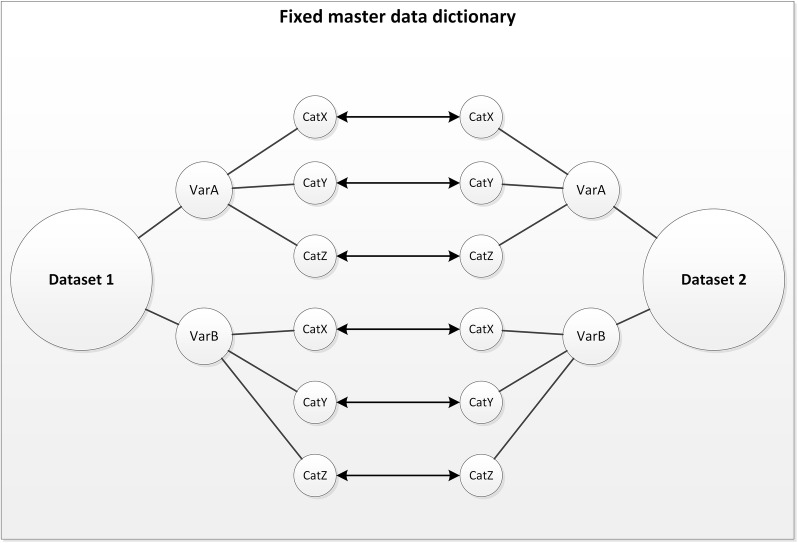
Harmonization model with a fixed master data dictionary. In a harmonization model with a fixed master data dictionary, single study’s data dictionary are adjusted and harmonized (arrow lines) to a master data dictionary that defines similar data from all studies

**Fig. 2:**
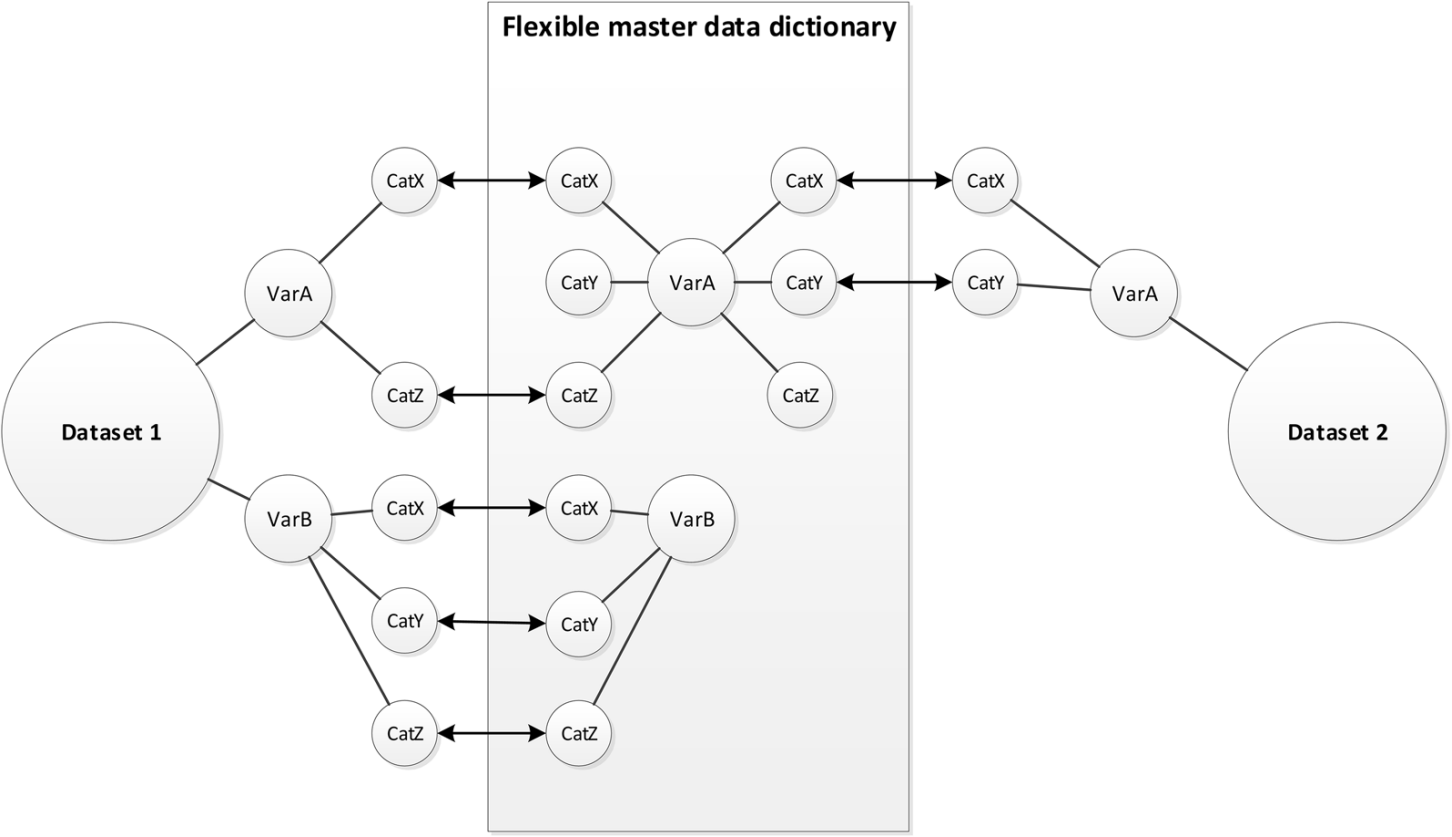
Harmonization model with a flexible master data dictionary. In a harmonization model with a flexible master data dictionary, the original study variables are harmonized on each category (arrow lines) with a master data dictionary that can be adapted

Different strategies can be used to harmonize IPD from multiple studies. Data can be transformed from the original data dictionary (i.e. a codebook with descriptions of variable names and value labels, variable type, format, and missing values) [8] to a fixed master data dictionary that defines similar and overlapping data from all studies (Fig. 1). This fixed master data dictionary can be defined prospectively (before data collection) or retrospectively (after all data has been retrieved), each with their specific challenges. A prospectively defined master data dictionary is time consuming when certain variables are defined differently across studies. For example, if age was assessed as a continuous variable in most studies (e.g. age in years), but as a categorical variable (e.g. < 50 vs. ≥ 50 years) in a newly retrieved study, all previously retrieved study data need to be transformed into categorical data in order to harmonize the datasets. On the other hand, retrospectively defining a master data dictionary can only be done after data collection of all variables of interest of identified studies has been completed. However, when the number of variables and datasets is large, it is more time-efficient to start harmonizing the data as soon as IPD from the first studies have been received. This way, data analyses can start soon after data collection has been completed. This requires a flexible strategy to harmonize IPD, allowing adaptations when new studies and/or variables with different coding schemes are included (Fig. 2). This also allows to easily add new studies at a later point in time.

We built a flexible data harmonization platform (DHP) to harmonize IPD from multiple studies. The DHP was primarily built for the Predicting OptimaL cAncer RehabIlitation and Supportive care (POLARIS) study [9–11], in which we harmonized IPD from—so far—57 randomized controlled trials (RCTs) to conduct an IPD-meta-analysis to evaluate the effects of physical activity or psychosocial interventions on health-related quality of life in patients with cancer, and to identify moderators of the intervention effects [9]. The DHP is also adopted in two other studies in which IPD were used from observational studies [12, 13]. In this paper, we describe the development and use of the flexible DHP to facilitate harmonization of IPD for meta-analyses.

## Main text

We developed a DHP that had to meet the following requirements: (I) allowing IPD harmonization with a flexible approach, (II) having the ability to store data in a centralized and secured database server with large capacity, (III) being transparent and easy in use, and (IV) having the ability to export harmonized IPD and corresponding data dictionary to statistical programs such as SPSS [14], STATA [15], SAS [16] or RStudio [17].

For this article, we used examples from the POLARIS study as proof of concept for which the DHP was initially developed [9–11]. Currently, the database of POLARIS includes IPD from almost 10,000 patients from 57 randomized controlled trials [9–11].

### Infrastructure DHP

Microsoft Access was used as front-end application. The front-end application includes interfaces that directly communicate with users, and forwards requests to a back-end server to retrieve requested data or to perform a requested service. The back-end server that can be used for this application is a relational database management system, such as Microsoft Structured Query Language (SQL) Server or MySQL. For POLARIS, the front-end application is connected with Microsoft SQL Server 12.0. This server has been set up at the Amsterdam University Medical Center—location VUmc, Amsterdam, The Netherlands. The DHP is secured by user identifier and password, and accessible for POLARIS consortium members that are authorized by the POLARIS steering committee.

Microsoft Access was chosen for the front-end application because of its widespread availability, and its ability to link with data files of different statistical software packages and to transfer both the data and the corresponding data dictionary into multiple tables in the relational database management system. The front-end application is linked to the tables in the relational database management system using an open database connectivity that enables communication between the front-end application and the relational database management system. To improve performance of the front-end application, we created pass-through queries that run statements that select, insert, update, and delete information in the relational database management system.

### Software requirements

To function adequately, the DHP has specific software requirements. The following software must be installed on a local computer: Microsoft Access 2010 (or newer), and a relational database management system such as Microsoft SQL Server or MySQL. Furthermore, Microsoft Access uses multiple required references that enable the DHP to communicate with statistical software programs. The Microsoft Access references required for adequate function of the DHP are: Visual Basic For Applications, Microsoft Access 14.0 object library (or newer), Microsoft Visual Basic for Applications Extensibility 5.3, OLE Automation, System_Windows_Forms, Microsoft ActiveX Data Objects 2.5 Library, Microsoft Scripting Runtime, mscorlib.dll, System, Microsoft Office 14.0 Access database engine Object Library (or newer), and Microsoft Windows Common Controls 6.0 (SP6). For POLARIS, the DHP has been set up to import SPSS data files, as most data files in POLARIS were provided in SPSS format. This requires SPSS to be installed on a local computer, as well as the following references in Microsoft Access: SPSS Statistics Type Library, and SPSS Statistics Legacy Type Library.

### User interfaces of the data harmonization platform

**Fig. 3:**
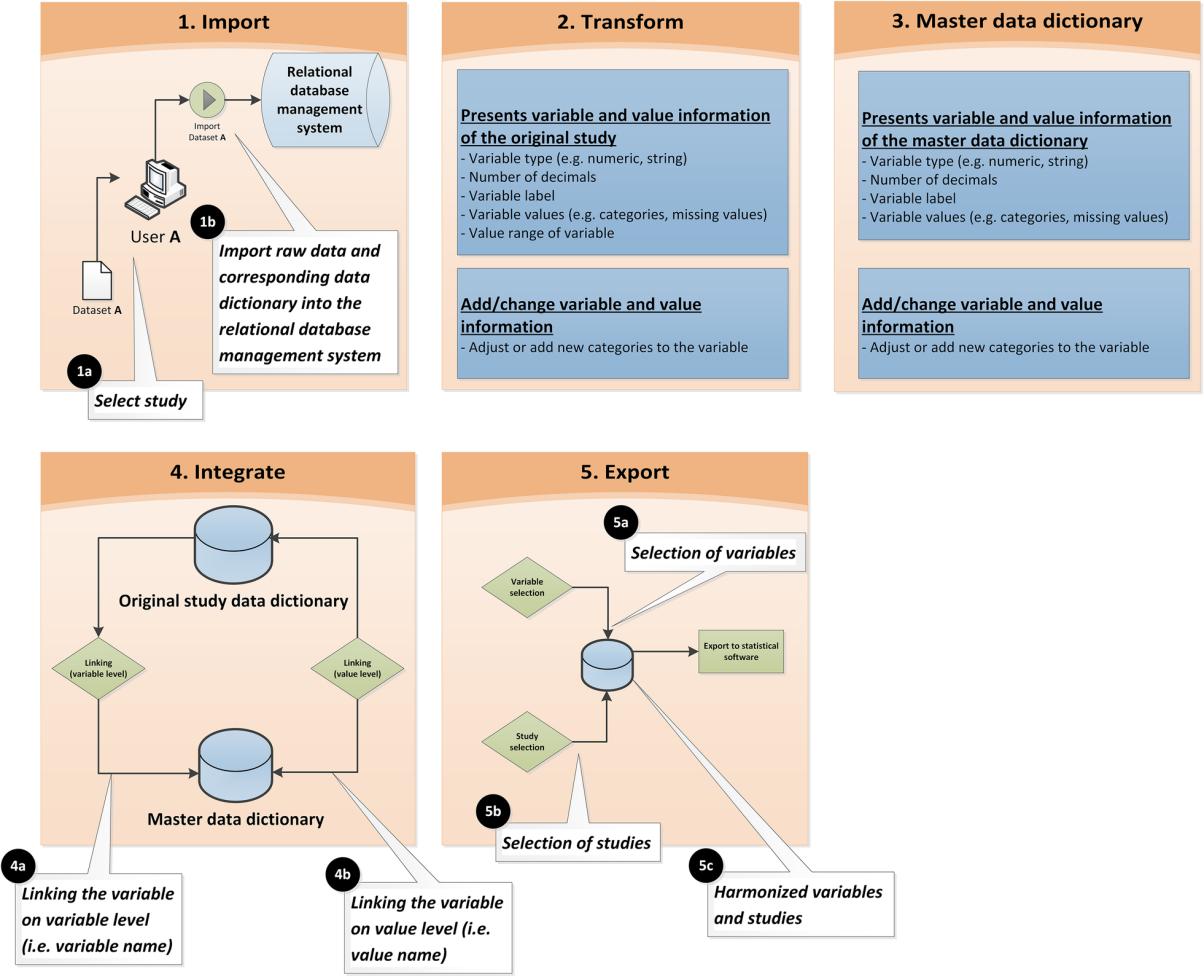
User interfaces of the data harmonization platform

The front-end application consists of five user interfaces, each with a separate function: (I) an import interface; (II) a transform interface; (III) a master data dictionary interface; (IV) an integration interface; and (V) an export interface (Fig. 3). These interfaces support the user with importing and harmonizing the data dictionary of the original study with the master data dictionary, and exporting the raw data of all selected variables and studies of interest into one harmonized dataset. The function of the five DHP user interfaces has been described in more detail in Additional file 1. A short description of the user interfaces is provided below.

#### Import

The import interface enables the user to select and import raw data and corresponding data dictionaries from original studies. The import of data is a fully automatic process in which raw data and corresponding data dictionaries are stored in predefined tables. The stored data includes study information (e.g. study name, source file pathname, import date of the study, and person responsible for the import of the study), variable information (e.g. variable name, type, labels, missing values, and study identifier), and value information (e.g. value definitions of categories and missing values (system and user) of specified variables, and study identifier). After being imported, the data is ready for transformation.

#### Transform

The transform interface shows the data dictionary from the original study, and presents the type (e.g. numeric, string), number of decimals, labels, values (i.e. categories and missing values), and value ranges (i.e. minimum and maximum value) of each variable. Accurately defining and labeling categories and missing values is essential to link the original study data dictionary with the master data dictionary [7]. Therefore, the transform interface enables users to make adjustments or to add new categories to the variables when necessary. For instance, missing values that are not defined as such, cannot be linked with the master data dictionary. Consequently, the data would incorrectly be imported as new numeric data into the harmonized dataset and not as missing data. This results in an incorrect outcome of the analyses, and therefore this transformation process is crucial to accurately harmonize the IPD into the new dataset.

#### Master data dictionary

The master dictionary interface shows the master data dictionary, and enables the user to add or adjust variables and/or categories in the master data dictionary during the data collection of eligible studies. It further provides information about the types, labels, number of decimals, and values of the variables in the master data dictionary and enables the user to adjust this information if necessary.

#### Integration

The integration interface enables the user to link the variable from the original study with the master data dictionary. The linking of variables occurs on the level of the variable itself (i.e. variable names) and on the value level (i.e. value definitions). When selecting the variables to be harmonized from the original study and the master data dictionary, the interface automatically shows the case missing values, and categories with the corresponding labels which can be linked. For study missing values, an algorithm has been created that adds study missing data (if needed) to the harmonized datasets when creating a harmonized dataset in the export interface. The integration interface further presents which variables from the original study are linked to the master data dictionary and which are not. Finally, it has the flexibility to disconnect linked specifications at the variable and/or value level, when a link was incorrect.

#### Export

The export interface enables the user to create a harmonized dataset from selected variables and from studies of interest in a preferred statistical software program. For POLARIS, we created harmonized datasets in SPSS. After selecting the preferred variables and studies, the user starts the fully automatic export process by pressing the ‘create file’ button in which the DHP runs an algorithm that creates a syntax in SPSS. Running this complete syntax creates a harmonized SPSS dataset including all selected variable names and studies that enables further analysis.

## Discussion

To the best of our knowledge, this is the first paper that describes a DHP that allows starting data harmonization already during data collection, which is time efficient, especially when the number of studies is large. It also allows adding data of new studies at a later point in time. With the increasing use of IPD meta-analysis [4], our flexible DHP helps managing the time necessary to harmonize IPD.

In contrast to previous DHPs for which all PI’s of original studies needed to transform their datasets to a defined master data dictionary before harmonization [7, 18], our DPH has the ability to store, prepare and harmonize IPD within one transparent DHP. The use of one centralized platform for data transformation, reduces the time burden for the PI of the original study. Our DHP is user-friendly, requiring minimal technical knowledge from the user. Instead of using syntaxes in statistical software [19], the harmonization process is facilitated by transparent interfaces, which are easy in use. Furthermore, our DHP enables the export of harmonized IPD and corresponding data dictionary to a statistical program of choice, creating more flexibility than offered in previous DHP where only R Software can be used [19].

To guarantee security of data, the DHP requires storage of the original datasets at one single secured location. To make explicit how and when the data is used, we have developed data sharing agreements for data access, use, and intellectual property arrangements for the POLARIS study [9]. Additionally, only fully anonymous datasets are shared by the PI’s of the original studies to ensure privacy of study participants [20].

Overall, the flexible DHP described in this paper facilitates harmonization of IPD already during the process of collecting data from multiple studies, allows to store, prepare, and harmonize IPD within one transparent platform, is easy in use, and has the ability to export harmonized IPD and corresponding data dictionary to a statistical program for further analysis. The DHP is currently being used in enriching the POLARIS study with data of new RCTs, and in two other IPD meta-analyses [12, 13].

## Limitations

The DHP is currently limited to import and export data files that are in SPSS format only. Exporting data to other statistical analyses software formats, such as SAS, STATA or R(Studio), can be possible, but additional algorithms have to be written first. Furthermore, the possibility to harmonize the IPD always depends on the measurement instruments and their measurement units used in the original studies to assess a certain construct. Therefore, to optimize harmonization process in the POLARIS study, we asked the PI of original study to share their data as ‘raw’ as possible.

## Additional file

**Additional file 1.** Detailed description of the five DHP user interfaces.

## Abbreviations

DHP: data harmonization platform
IPD: individual patient data
PI: principal investigator
POLARIS: Predicting OptimaL cAncer RehabIlitation and Supportive care
RCT: randomized controlled trial
SAS: statistical analysis system
SPSS: Statistical Package for the Social Sciences
SQL: Structured Query Language
