# Supplementary material for: Development and use of a flexible data harmonization platform to facilitate the harmonization of individual patient data for meta-analyses
Source: BMC Res Notes. 2019 Mar 22;12:164. doi: 10.1186/s13104-019-4210-7 (PMC6431032; doi:10.1186/s13104-019-4210-7)
Supplement: Supplementary file 1 — Additional file 1. Detailed description of the five DHP user interfaces. [file 13104_2019_4210_MOESM1_ESM.docx]

**Function of the five DHP user interfaces**

***Import***

In the import interface, the user selects the dataset from the original study and starts the import process by pressing the import button. The import of data is a fully automatic process that includes the following five steps:

In the first step, the DHP imports the raw data into a newly defined table in the relational database management system. In the second step, the DHP stores the data dictionary from the original study in three empty temporary data dictionary tables: one including study information (e.g. study name, source file pathname, import date of the study, and person responsible for the import of the study), one including variable information (e.g. variable name, type, labels, missing values, and study identifier), and one including value information (e.g. value definitions of categories and missing values (system and user) of specified variables, and study identifier). In the third step, the DHP compares the study, variable names, and value definitions of the imported study stored in the temporary data dictionary tables, with those stored in three identical structured permanent data dictionary tables. Comparing the temporary data dictionary tables with the permanent tables is a fully automatic process that distinguishes four differences:

1. The original study that is included in the temporary data dictionary table has no corresponding study identifier in the permanent data dictionary table. The DHP recognizes this as a new study, and adds the original study with associated variable names and value definitions and corresponding variable names and value definitions (e.g. types, number of decimals, labels, categories, and missing values) to the permanent data dictionary tables.
2. The original study has previously been included in the DHP (i.e. information about the original study is in the permanent data dictionary table), but the variable names and/or value definitions included in the temporary data dictionary table(s) have no corresponding variable names and/or value definitions in the permanent data dictionary table(s). The DHP identifies this as a new variable name and/or value definitions in the original study, and adds the variable name and/or value definitions along with the corresponding variable names and/or value definitions to the permanent data dictionary table(s).
3. The original study has previously been included in the DHP, and although the variables names and/or value definitions in the temporary data dictionary table(s) match those in the permanent data dictionary table(s), the variable and/or value information in the temporary data dictionary table(s) does not correspond with the variable and/or value information in the permanent data dictionary table(s).
4. The original study has previously been included in the DHP, but the variable names and/or value definitions that are included in the permanent data dictionary table(s) have no corresponding variable names and/or value definitions in the temporary data dictionary table(s). The DHP recognizes this as a deleted variable name and/or value definition from the original study, and inactivates the variable name and/or value definition in the permanent data dictionary table(s). Consequently, the user cannot harmonize these variables and/or values.

In the fourth step, the DHP imports the raw data from the original study into an entity-attribute-value table. This table consists of unique rows where each attribute-value pair describes one attribute of a given entity. The entity represents a subject identifier of an original study, for example ‘232’ in case the subject identifier from the original study is 232. The attribute represents an entity, for example the variable ‘Age’. The value is the value of that attribute, for example ‘59’ in case the age is 59 years.

In the final step, the DHP produces a table containing the 5 highest and the 5 lowest values, including system missing values, of all variables to inform the user in the transform interface about the value range of each variable.

***Transform***

In the transform interface, the user manually selects a variable name from the original study that he or she wants to check and prepare for linking with the master data dictionary. First, the user checks if the label clearly describes the corresponding variable (e.g. patient’s age in years at baseline, Figure 1). Defining the label is essential for linking the correct variable name with the corresponding variable name in the master data dictionary. Next, the user checks if the variable is a continuous or a categorical variable. Categories and missing values need to be linked as categories with the master data dictionary and should therefore be described. If categories and missing values are not described, the user can add the value definition identifying the category and missing value by using the “add value to variable” button (Figure 1).

Figure S1. Screenshots from the transform interface where the data dictionary of the original study is presented (in the ‘Current Value’ grey fields). It presents the user (1) the variable type (e.g. numeric), number of decimals (e.g. ‘0’), and label (e.g. ‘Age’), (2) values (i.e. categories, and user (‘9999’) and system missing (‘SYSMIS’) values) of the variable, and (3) value ranges (i.e. five highest and five lowest values) of the variable. It further enables the user to make adjustments to the variable and value information and to add new categories to the variables when necessary (in the ‘New Value’ white fields).


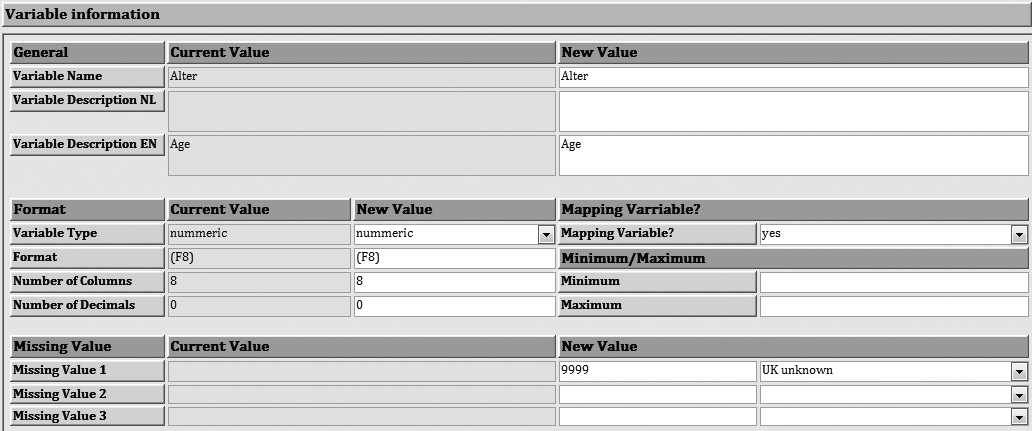


1


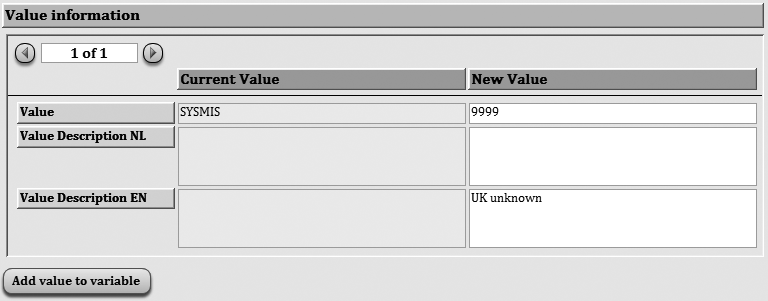


2


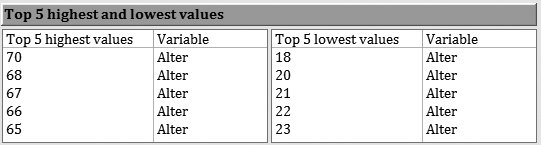


3

***Master data dictionary***

In the master data dictionary interface, the user has insight in all variable names and value definitions with the corresponding information described in the master data dictionary, including, among others, the type (e.g. continuous, string, or categorical), the number of decimals, and values (i.e. categories and missing values) of the variable. It does, however, not present which variable names and/or value definitions have been linked with the corresponding variable names and/or value definitions from the original studies.

***Integration***

In the integration interface, the user selects variables from the original study that need to be linked with the master data dictionary. For example, in the POLARIS study, we linked the variables ‘Alter’ (German for ‘age’) and ‘Geschl’ (abbreviation for geschlacht, which is German for sex) from a German study with the master data dictionary (Figure 2). The variable ‘Alter’ is a continuous variable representing the age of a patient at baseline. The variable ‘Geschl’ is a categorical variable that represents the sex of a patient, with the value ‘1’ representing male and ‘2’ representing female.

In order to harmonize these variables with the master data dictionary, the user performs several steps:

First, the user selects the variable ‘Alter’ or ‘Geschl’ from the original study to be harmonized. Subsequently, the interface automatically shows the corresponding value definitions in a value list. As value definitions from continuous variables do not differ between studies (i.e. the value ‘59’ for a patient’s age is similar across studies), only value definitions from categorical and missing values need to be linked with the master data dictionary. Consequently, when selecting the variable ‘Alter’, the interface only shows the codes and labels for missing values (i.e. the value code ‘9999’ with a corresponding label ‘Unknown’, Figure 2). When selecting the variable ‘Geschl’, the interface shows the missing values, and categories with the corresponding labels (i.e. the value definitions ‘1’ and ‘2’ with the corresponding labels ‘male’ and ‘female’, respectively).

Second, the user selects a variable name to be harmonized from the master data dictionary that corresponds with the selected variable from the original study. For example, the variable ‘Alter’ is described in the master data dictionary as ‘Age’ with the label ‘Age (years)’. After selecting the variable ‘Age’ from the master data dictionary, the integrate interface automatically shows the corresponding values with labels from this variable (i.e. the value ‘9999’ with label ‘Unknown / do not know’). In case the variable ‘Age’ and/or the missing value definitions are not described in the master data dictionary, the user opens the master data dictionary interface and adds the variable ‘Age’ in the master data dictionary using the “add variable” button. Next, the user describes the variable information of ‘Age’ (e.g. continuous variable), and adds the missing value definition ‘9999’ with label ‘Unknown’ to define the missing value by using the “add value” button.

Third, the user links the variable ‘Age’ with the variable ‘Alter’ on two levels; on variable name (i.e. ‘Alter’ is linked with ‘Age’ from the master data dictionary), and on value definition (i.e. the missing value ‘9999’ with label ‘Unknown’ from ‘Alter’ is linked with the missing value ‘9999’ with label ‘Unknown / do not know’ from ‘Age’, Figure 2). The variable ‘Geschl’ from the original study is linked with ‘Sex’ from the master data dictionary. On value level, the definitions ‘1’ with label ‘male’, ‘2’ with label ‘female’, and ‘9999’ with label ‘Unknown’ from the variable ‘Alter’ are linked with ‘0’ with label ‘male’, and ‘1’ with label ‘female’, and ‘9999’ with label ‘Unknown / do not know’ from ‘Sex’, respectively.

Figure S2. Screenshots from the integrate interface that enables the user to link the variable of the original study with the master data dictionary. The linking of variables occurs on (1) the level of the variable itself (i.e. variable names) and (2) on the value level (i.e. value codes). It has further the flexibility to disconnect linked specifications at the variable and/or value level using the arrow buttons, when, for example, a link was incorrect.


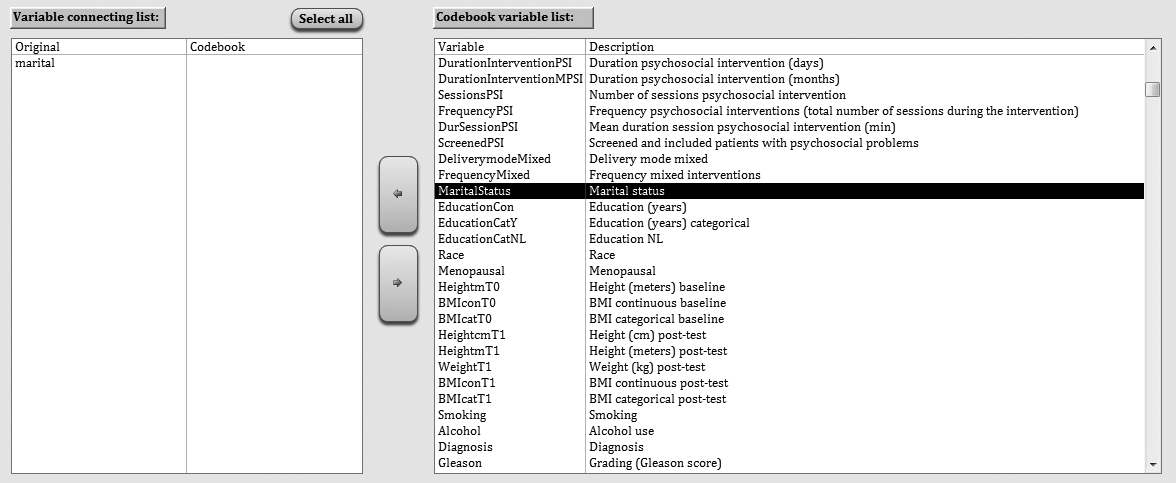


1


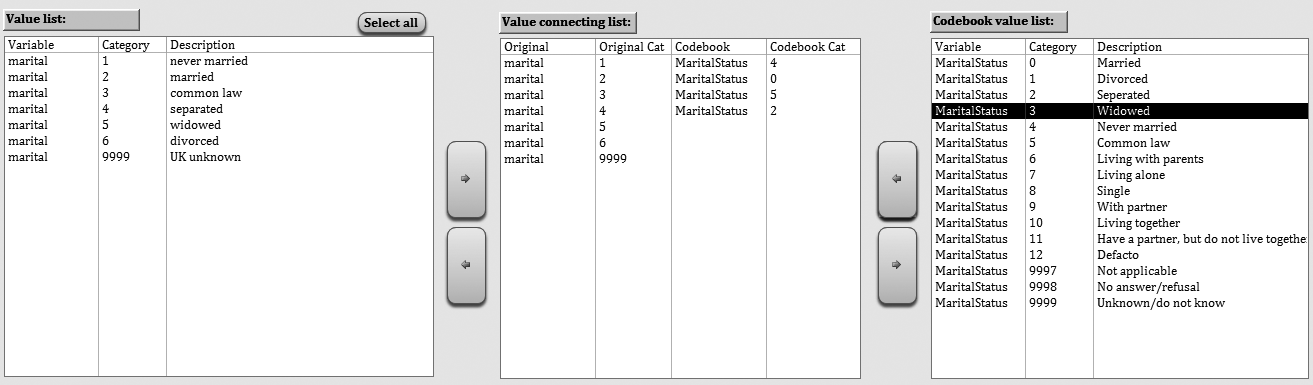


2

***Export***

In the export interface, the user creates a harmonized dataset in a preferred format to be able to proceed with the proposed statistical analyses. For POLARIS, we created harmonized datasets in SPSS. The export process includes the following steps:

First, the user selects variable names to be harmonized from the variables presented in the master data dictionary. For example, the user selects the variable names ‘Age’ and ‘Sex’. Second, the user selects the studies to be harmonized from all the imported studies that are presented in the master data dictionary study identifier. For example, the user selects the three imported studies with study identifier ‘6’, ‘8’, and ‘15’. Third, the user starts the fully automatic export process by pressing the ‘create file’ button. In this process, the DHP combines all raw data from the selected variables and studies – with the corresponding data dictionary as described in the master data dictionary – into a newly defined table in the relational database management system (i.e. presented as ‘tblExport’, Table 1).

**Table S1.** Example of the table ‘tblExport’ where all the raw data of the selected variables and studies with the corresponding variable name (‘VarCode’) and value definition (‘ValDef’) as described in the master data dictionary.

| **SubjId** | **StudyId** | **CountryId** | **VarCode** | **ValDef** |
| --- | --- | --- | --- | --- |
| 600232 | 6 | 1 | Age | 59 |
| 600232 | 6 | 1 | Sex | 1 |
| 800056 | 8 | 7 | Age | 67 |
| 800056 | 8 | 7 | Sex | 0 |
| 150101 | 15 | 10 | Age | 54 |
| 150101 | 15 | 10 | Sex | 1 |
| … | … | … | … | … |

Abbreviations: SubjId= subject identifier; StudyId= study identifier; CountryId= country identifier.

This table has a long format, where each row in this example represents a variable name (e.g. ‘Age’) and value definition (e.g. ‘59’) of a newly created subject identifier from the original study (e.g. ‘600232’ has been created from the original subject identifier ‘232’ and the subject’s related study identifier ‘6’, Table 2).

Table S2. Example of a restructured dataset in SPSS that has been reshaped from a long data file (see Table S1) into a wide data file.

| **SubjId** | **StudyId** | **CountryId** | **Age** | **Sex** |
| --- | --- | --- | --- | --- |
| 600232 | 6 | 1 | 59 | 1 |
| 800056 | 8 | 7 | 67 | 0 |
| 150101 | 15 | 10 | 54 | 0 |
| … | … | … | … | … |

Abbreviations: SubjId= subject identifier; StudyId= study identifier; CountryId= country identifier.

Each row also provides information on the subject’s related study identifier (e.g. ‘6’), the rank of the variable (i.e. the ranking order of the variable name column in the exported dataset), and the country identifier (e.g. the country identifier ‘1’ represents ‘The Netherlands’). Next, the DHP runs an algorithm that creates a syntax in a statistical software program (e.g. SPSS). In this syntax, the DHP copies five commands that creates a dataset from the raw data of the ‘tblExport’ into the statistical software program:

1. A command to import the data stored in the ‘tblExport’ into the statistical software program. For POLARIS, SPSS retrieves the data from the ‘tblExport’ using the ‘get data’ command. With this command, SPSS selects the data stored in the ‘tblExport’ via an open database connectivity and import the data into a new defined SPSS dataset.
2. A command to restructure the imported data from a long format into a wide format. In SPSS, the data are restructured by the ‘casestovars’ command. With this command, the data stored in the ‘tblExport’ are reshaped, making one row per subject identifier that would contain the subject’s related study identifier, the country identifier, ‘Age’, and ‘Sex’ as variables (Table 2).
3. A command to set variables that are not included for some studies into study missing. For example, if the variable ‘Age’ is not included in a study, all values of ‘Age’ within this study is set to the missing value ‘9997’ with label ‘Study missing’.
4. A command to set the data dictionary for each corresponding variable in the newly created dataset. For example, the variable ‘Age’ is set to a continuous variable, with two decimals, and has ‘9997’ with label ‘Study missing’, ‘9998’ with label ‘not applicable (N/A)’, and ‘9999’ with label ‘Case missing’ as missing values. The variable ‘Sex’ is set to a categorical variable, has ‘0’ with label ‘male’ and ‘1’ with label ’female’ as categories, and has ‘9997’ with label ‘Study missing’, ‘9998’ with label ‘N/A’, and ‘9999’ with label ‘Case missing’ as missing values.
5. A command that saves the data file into a specified folder. For POLARIS, the data files are stored on a secured server that is only accessible for authorized POLARIS consortium members.

Running the complete syntax creates a harmonized SPSS dataset including all selected variable names and studies that enables further analysis.
